# Annotations

**Published:** 1903-09-19

**Authors:** 


### ANNOTATIONS.

### The Skull and the Brain.

The address delivered by Professor Johnson Symington, as President of the Anthropological Section of the British Association, is one of considerable interest. Whilst not announcing any commanding fact or particularly novel theory, it points out the line along which future study of the functions of the several parts of the brain may be profitably pursued. There are two positions related to this subject fairly within the grasp of modern knowledge. First, the racial peculiarities of the form and size of the skull are known on the basis of an exact and complete investigation. It is also generally recognised that whilst the chief factor in determining cranial form is brain growth, there are other influences which contribute to this end. Hence the mere study of cranial shape and capacity offers only a limited possibility of knowledge in reference to cerebral development and function. Secondly, it is well known that definite areas of the cerebral cortex are connected with the actions of various groups of muscles, and that other areas are associated with the reception of nerve impulses from the peripheral sense organs. But these areas occupy only a limited portion of the surface of the cerebral cortex. The greater part of the cortex is a *terra incognita*. There exists no definite information regarding the localisation of mental and moral qualities. Hence what is wanted is not merely a knowledge of the specific peculiarities of the skull in the various races and classes of mankind, for these afford no certain criteria regarding brain development. It is the actual brain facts which are required. When these are fully known there will be some prospect of arguing from them, and from the respective mental and moral qualities of the different races, to definite conclusions regarding the functions of those parts of the cerebral cortex which at present are shrouded in physiological darkness.

### Education and Infectious Diseases.

A great deal has been said and written to the end that fresh legislation is needed on matters affecting public health in order that the nation may reap the benefits of recent advances in medicine, especially such as bear upon the prevention of disease. Doubtless there is a good deal that can be urged in favour of such a contention, but it is well to bear in mind the possibility that new regulations may outstrip public morality. In such a case there will be an inevitable neglect of the statute in question and a corresponding loss of respect for the law as a whole. The point is exemplified by what happened at Chicago during a recent outbreak of scarlet fever when, it is stated, among others the following vigorous precautionary steps were taken :—No children were allowed in the streets ; all visiting was prohibited ; all public gatherings in churches, Sunday schools, and lodges, and parties were positively interdicted, and, as an extra precaution, all stray dogs and cats were ordered to be shot on sight. A house-to-house inspection was instituted and all who had sore throats were ordered to report the fact to some physician. Every case was promptly isolated and the home placarded, and after the patient was well again the place was thoroughly fumigated by the public disinfector, who was empowered to burn all mattresses and whatever he deemed it wise to destroy. Certainly the public health officials were by no means supine here, but note the effect of all this rigour ; the epidemic lingered on because poor, weak human nature found it very tiresome to act up to the high official standard set for it and therefore walked in public places with the rash in full bloom and took especial pains to conceal the fact of its illness from the officials. It is clear that if legislation is to be effective in public health affairs it must not be of a too rigorous nature and it must proceed side by side with education.

### The National Food Bill.

Sir Robert Giffen in an interesting and instructive paper on "The Wealth of the Empire, and how it should be Used," read before the British Association last week, estimated our national expenditure on food and drink at nearly 600 millions sterling, and not unreasonably described the amount as one which naturally gave rise to the inquiry whether in some directions there might not be a possibility of retrenchment to the advantage of the community. He quoted a former estimate, presented to the Association about twenty years ago by a committee comprising Mr. Jevons, Mr. Leone Levi, and other distinguished authorities, in which it was alleged that the inhabitants of these islands spent 1·5 per cent. of their total income on tobacco, as against 1·3 per cent. on education, 1·4 per cent. on church, 0·8 per cent. on literature, 0·6 per cent. on newspapers, and 0·7 per cent. on theatres, music-halls, and other amusements. There can be no doubt, whether the foregoing estimates were correct or not, that the proportion devoted to tobacco is vastly greater now than it was 20 years ago ; and it is difficult to believe that the increase can have been beneficial to the consumers. On the general question, as Sir Robert pointed out, it is necessary to inquire whether there may not be a consumption of meat and alcohol among the artisans and wealthier classes in excess of what is required for the maintenance of health and strength ; an excess which must be at once an economic waste and a source of ill-health by its effect upon the overburdened organs of digestion and elimination. On the other hand, there is good reason to fear that, in spite of the magnitude of the total expenditure, its distribution is painfully unequal, and that large numbers of people are insufficiently fed. Here, as in so many other directions, we see how much room there is for bringing the elements of physiology to bear upon the work which is conducted under the more or less specious name of education. It is an old and familiar remark, but is none the less perfectly true, that the waste of an ordinary English family would suffice to maintain an ordinary French family of the same rank and number in health and comfort ; and the difference is one which might at least be remedied by education. The knowledge how to select the materials of a meal, and how to present them in wholesome and economical forms, is more likely to be useful to a Board School girl than a good deal of the information which is now suffered to pass through her head.

